# Diagnostic performance of microRNAs for predicting response to transarterial chemoembolization in hepatocellular carcinoma: a meta-analysis

**DOI:** 10.3389/fonc.2024.1483196

**Published:** 2025-01-14

**Authors:** Tianyi Huang, Jing Chen, Lu Zhang, Rui Wang, Yiheng Liu, Cuihua Lu

**Affiliations:** ^1^ Department of Gastroenterology, Affiliated Hospital of Nantong University, Medical School of Nantong University, Nantong, China; ^2^ Medical School of Nantong University, Nantong, China

**Keywords:** miRNAs, TACE, diagnostic accuracy, meta-analysis, HCC

## Abstract

**Purpose:**

To provide a detailed pooled analysis of the diagnostic accuracy of microRNAs (miRNAs) in predicting the response to transarterial chemoembolization (TACE) in hepatocellular carcinoma (HCC).

**Methods:**

A comprehensive literature search was conducted across PubMed, Embase, Cochrane Library, and Web of Science to identify studies assessing the diagnostic performance of miRNAs in predicting TACE response in HCC. Two independent reviewers performed quality assessment and data extraction using the Quality Assessment of Diagnostic Accuracy Studies (QUADAS-2) tool. Pooled sensitivity, specificity, positive likelihood ratio (PLR), negative likelihood ratio (NLR), diagnostic odds ratio (DOR), and the area under the summary receiver operating characteristic (SROC) curve were calculated using a bivariate random-effects model. Subgroup analyses and meta-regression were performed to explore potential sources of heterogeneity, including sample size, response criteria, specimen source, response evaluation methods, TACE efficacy interval window, and geographical location.

**Results:**

Seven studies, comprising 320 HCC responders and 187 non-responders, were included in this meta-analysis. The miRNAs studied included miR-373, miR-210, miR-4492, miR-1271, miR-214, miR-133b, and miR-335. The pooled sensitivity of miRNAs in predicting recurrence after TACE was 0.79 [95% CI: 0.72-0.84], and the pooled specificity was 0.82 [95% CI: 0.74-0.88]. The DOR was 17 [95% CI: 9-33], and the pooled area under the SROC curve (AUC) was 0.85 [95% CI: 0.81-0.88], indicating excellent diagnostic accuracy. Subgroup analyses revealed significant differences in diagnostic performance based on response criteria and geographical location. Meta-regression did not identify any significant sources of interstudy heterogeneity.

**Conclusion:**

MiRNAs show promise as diagnostic tools for predicting TACE response in HCC patients. However, their clinical application requires further validation in larger cohorts. Future research should focus on standardizing RNA extraction methods, selecting consistent endogenous controls, and adopting uniform response evaluation criteria to improve reliability and reduce variability.

## 1 Introduction

Hepatocellular carcinoma (HCC) is one of the most common and deadly types of liver cancer, significantly contributing to cancer-related deaths globally (1). The outlook for HCC patients largely depends on how early the cancer is detected and the effectiveness of the treatments available (2). Among the various treatment options, transarterial chemoembolization (TACE) has become a key therapy for intermediate-stage HCC. TACE involves the local delivery of chemotherapy combined with embolization to cut off the tumor’s blood supply, enhancing drug retention and effectiveness (3). Despite its extensive use, responses to TACE vary greatly, with many patients experiencing poor outcomes (4). Therefore, finding predictive biomarkers to accurately predict the response to TACE is crucial (5). Recently, microRNAs (miRNAs) have attracted considerable interest as potential biomarkers for various cancers, including HCC (6, 7). miRNAs are small, non-coding RNA molecules, typically 18-25 nucleotides long, that regulate gene expression post-transcriptionally (8). They are vital in numerous cellular processes such as cell proliferation, differentiation, apoptosis, and metastasis. The dysregulation of miRNAs is linked to the development and progression of HCC, making them promising candidates for diagnostic, prognostic, and predictive biomarkers (9). Several studies have investigated the potential of miRNAs to predict the response to TACE in HCC patients (10). However, results have been inconsistent, with differences in miRNA profiles, sample sizes, and methodologies (10–13). This inconsistency highlights the need for a comprehensive review of the existing evidence to clarify the diagnostic performance of miRNAs in this context. This meta-analysis aims to systematically assess the diagnostic accuracy of miRNAs in predicting the response to TACE in HCC patients. By combining data from multiple studies, we aim to provide robust estimates of sensitivity, specificity, diagnostic odds ratio (DOR), and the area under the summary receiver operating characteristic (SROC) curve. We also intend to identify sources of variability and evaluate the impact of study design, miRNA profiling techniques, and other factors on diagnostic performance. The findings from this meta-analysis could significantly impact the clinical management of HCC. Demonstrating high diagnostic accuracy of miRNAs could lead to their incorporation into clinical practice, improving patient stratification and treatment outcomes. Additionally, understanding the limitations and sources of variability in current research could guide future studies toward more standardized and reliable approaches. In summary, this meta-analysis aims to provide a thorough and rigorous evaluation of the diagnostic performance of miRNAs in predicting the response to TACE in HCC patients. By doing so, it seeks to offer valuable insights that could inform clinical decision-making and enhance the prognosis for HCC patients undergoing TACE.

## 2 Materials and methods

This meta-analysis was carried out in accordance with the 2020 guidelines specified by the PRISMA-DTA (Preferred Reporting Items for Systematic Reviews and Meta-Analyses of Diagnostic Test Accuracy) statement (14).

### 2.1 Literature search

Two independent authors conducted a systematic literature search across PubMed, Embase, Cochrane Library, and Web of Science using the keywords: (HCC) AND (TACE) AND (miRNA). The search was finalized on July 19, 2024 without any limitations on the language or date of publication.

### 2.2 Inclusion criteria

The inclusion criteria were established as follows: (1) patients diagnosed with HCC pathologically; (2) HCC cases treated with TACE; (3) assessment of miRNA expression in both responder and non-responder groups; (4) sufficient data to create 2 × 2 diagnostic tables for diagnostic studies; or (5) availability of area under the curve (AUC) data along with the number of responders and non-responders.

### 2.3 Exclusion criteria

The exclusion criteria included: (1) duplicate publications; (2) case reports, letters, reviews, editorials, meeting abstracts, and animal studies; (3) studies not relevant to the topic; (4) studies without complete data for analysis; (6) studies in languages other than English; and (7) studies from a single institution where similar data had already been published. The study selection was carried out by two independent authors, and any disagreements were resolved through consensus.

### 2.4 Data extraction

The following information from the selected literature was extracted: miRNA name, author name with publication year, country, miRNA detection method, number of cases and controls, AUC, sensitivity and specificity of each miRNA. We obtained numerical information for the meta-analysis, including a (2 × 2) contingency table consisting of true positive (TP), false negative (FN), true negative (TN), and false positive (FP) values. Two researchers independently extracted the study data, resolving any disagreements through discussion until reaching a consensus.

### 2.5 Quality assessment

In this meta-analysis, the Quality Assessment of Diagnostic Accuracy Studies (QUADAS-2) tool was utilized to evaluate the methodological quality of the included studies. QUADAS-2 assesses four key domains: patient selection, index test, reference standard, and flow and timing, categorizing the risk of bias as high, low, or unclear. Two researchers independently conducted the assessments using RevMan 5.4 software, resolving any discrepancies through discussion to achieve consensus.

### 2.6 Statistical data analysis

The meta-analysis was conducted using the “MIDAS” module in STATA version 15.0, OpenMeta[Analyst], Meta-Disc software, and meta4diag package in R. The diagnostic value of MicroRNA was evaluated through the SROC plot and AUC. Key metrics such as pooled sensitivity, specificity, positive likelihood ratio (PLR), negative likelihood ratio (NLR), DOR, and 95% confidence intervals (CIs) were computed using a random effects model. Meta-regression and subgroup analyses were carried out to investigate the sources of heterogeneity. The overall diagnostic performance was measured with the SROC curve and AUC, considering a significance threshold of p-value < 0.05. Heterogeneity was assessed using Cochran’s Q test and Higgins’ I^2^, with an I^2^ > 50% indicating substantial heterogeneity. Publication bias was checked with a Deeks’ funnel plot, and statistical significance was evaluated using the Deeks’ asymmetry test. Sensitivity analysis was conducted using the leave-one-out method in Open Meta-Analyst software. A p-value < 0.05 was considered significant for all tests.

## 3 Results

### 3.1 Literature search and selection of studies

**Figure 1:**
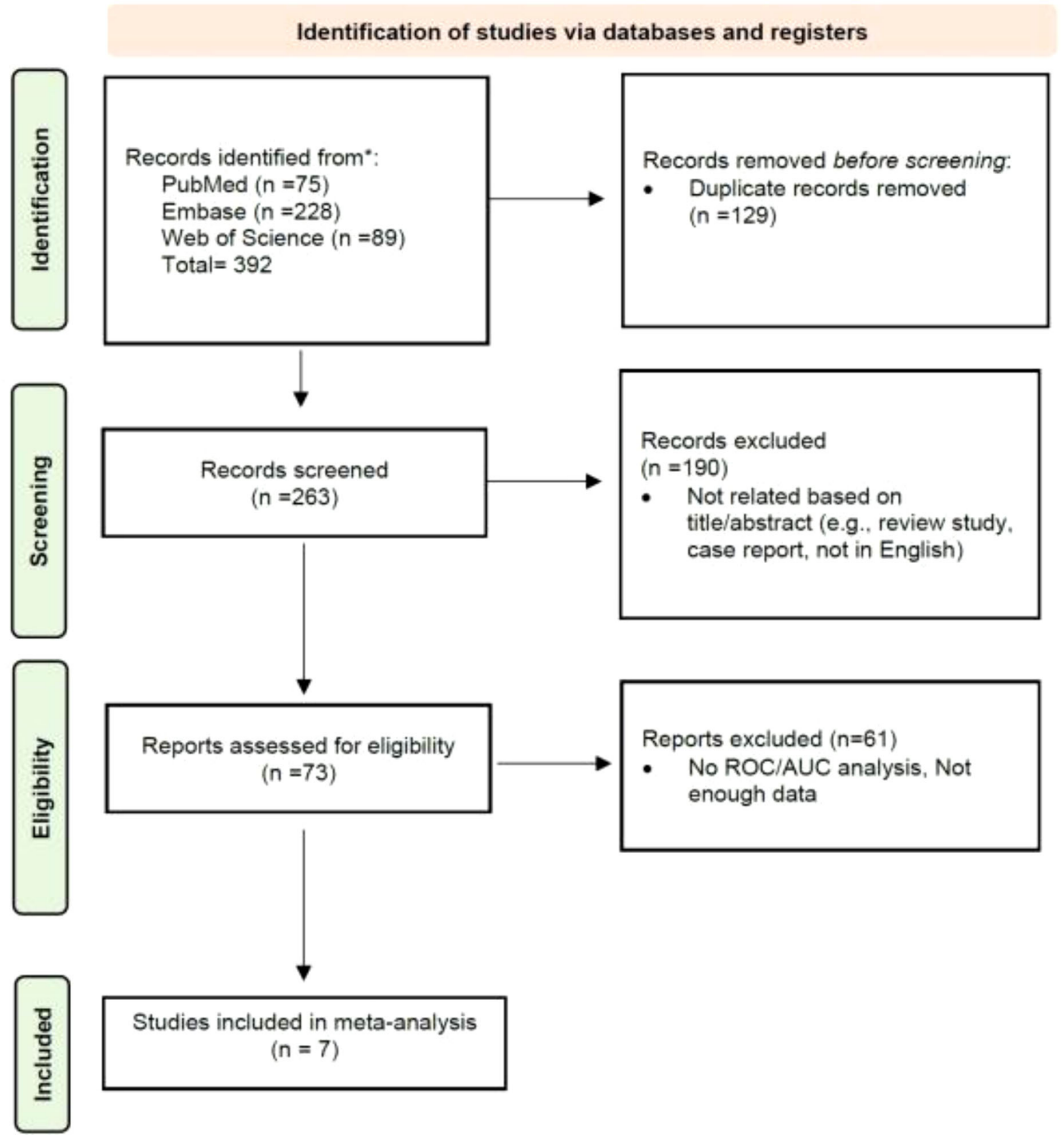
PRISMA flowchart of the study selection process.

A thorough database search initially identified 392 records. After the removal of 129 duplicate entries, 263 unique titles remained for further assessment. Screening of these titles and abstracts led to the exclusion of 190 papers deemed irrelevant to the research focus, such as review articles, case reports, or publications in languages other than English. Following a more detailed review, an additional 66 citations were excluded based on specific inclusion and exclusion criteria. Consequently, 7 studies were selected for inclusion in the meta-analysis. The detailed study selection process is presented in **Figure 1**.

### 3.2 Characteristics of the included studies

**Table 1** summarizes the studies that used miRNAs as biomarkers for assessing the response to TACE in patients from different countries, focusing on the performance of these miRNAs in distinguishing between responders and non-responders to TACE treatment (10–12, 15–18). These studies cover diverse geographic locations, including Egypt (12, 17), China (11, 15, 16, 18), and Italy (10), reflecting a broad interest in miRNA research across different populations. The sample sizes across the studies vary significantly, with the number of cases (both responders and non-responders) ranging from a minimum of 40 to a maximum of 162 patients. The miRNAs examined include miR-373, miR-210, miR-4492, miR-1271, miR-214, miR-133b, and miR-335. Among these, miR-210 and miR-373 were upregulated, acting as oncogenes in HCC patients; however, their expression levels decreased following TACE treatment. In contrast, other miRNAs exhibited an opposite trend. The performance of miRNAs, as measured by the AUC, varies as well, highlighting the differential diagnostic power of each miRNA. For instance, Salah El-Din Tork (12) in Egypt studied miR-373, reporting an AUC of 0.767. In contrast, Pratama et al. (10) in Italy focused on miR-4492, with a higher AUC of 0.84. The studies also vary in their treatment regimens. For example, Salah El-Din Tork’s (12) study used doxorubicin, while Ali et al.’s (17) used cisplatin and doxorubicin. Other studies did not specify the TACE regimen used, highlighting a degree of variability in treatment protocols. Regarding biological samples, most studies used serum as the source for miRNA extraction, except for Salah El-Din Tork’s (12) study, which used plasma. The RNA extraction methods and reverse transcription techniques also differ across studies. The miRNeasy Mini Kit and miRNA miScript II RT Kit were used in Salah El-Din Tork’s (12) study, while other studies employed kits like Trizol (15), Agilent Small RNA kit (10), and miR VanaTM (11). The diversity in methodologies reflects the evolving nature of miRNA research and the adaptation of different protocols to optimize results. The criteria for response assessment and follow-up periods also show variation. The majority of studies used the modified Response Evaluation Criteria in Solid Tumors (mRECIST) to evaluate response, ensuring a standardized measure of treatment efficacy. However, Ali et al.’s (17) study used RECIST criteria, and follow-up periods ranged from 4-6 weeks to 3 months, with some studies not specifying the follow-up duration (10, 16, 17). The basis for defining responders and non-responders also varied: some studies, like Salah El-Din Tork’s (12), defined response as complete response (CR) plus partial response (PR) versus non-responders (NR), while others, like Pratama et al. (10), compared complete responders (CR) to non-responders (NR) and partial responders (PR) grouped together.

**Table 1:** Characteristics of the included studies.

Study	Country	miRNA name	AUC	Cases	Regulation in Responders	Regulation in HCC Patients	TACE Regimens	Source	Reverse Transcription	RNA ExtractionMethod	Criteria for Response	Follow Up Period	Ref.
Salah El-Din Tork 2023	Egypt	miR-373	0.767	45 Responders 8 Non-Responders (CR+PR) vs. NR	↓	↑	Doxorubicin (50 mg)	Plasma	miRNA miScript II RT Kit	miRNeasy Mini Kit	mRECIST	3 Months	(12)
You 2021	China	miR-210	0.698	26 Responders 14 Non-Responders (CR+PR) vs. NR	↓	↑	–	Serum	RT-qPCR	Trizol	mRECIST	4-6 Weeks	(15)
Pratama et al., 2020	Italy	miR-4492	0.84	14 Responders 32 Non-Responders CR vs. (NR+ PR)	↑	↓	–	Serum	qRT-PCR	Agilent Small RNA kit	mRECIST	NM	(10)
Guo et al., 2020	China	miR-1271	NM	112 Responders 50 Non-Responders CR+PR+SD vs. Relapse	↑	↓	Fluorourea glycoside + Oxaliplatin	Serum	TaqMan qRT-PCR	miR VanaTM	mRECIST	3 Months	(11)
Tang et al., 2020	China	miR-214	0.849	56 Responders 31 Non-Responders (CR+PR+SD) vs. Relapse	↑	↓	–	Serum	qRT-PCR	Transgene RNA extraction kit	–	NM	(16)
Ali et al., 2019	Egypt	miR-133b	0.965	33 Responders 18 Non-Responders (CR+PR) vs. NR	↑	↓	Cisplatin (50 mg) and doxorubicin (50 mg)	Serum	miScript RT kit	miRNeasy kit	RECIST	NM	(17)
Cui et al., 2015	China	miR-335	0.922	34 Responders 91 Non-Responders (CR+PR) vs. NR	↑	↓	–	Serum	RT-PCR	QIAamp RNA Blood kit	RECIST	1 Month	(18)

AUC, Area Under the Curve; TACE, Transarterial Chemoembolization; CR, Complete Response; PR, Partial Response; NR, Non-Responder; SD, Stable Disease; RT-qPCR, Reverse Transcription Quantitative Polymerase Chain Reaction; qRT-PCR, Quantitative Reverse Transcription Polymerase Chain Reaction; miRNA, MicroRNA; mRECIST, Modified Response Evaluation Criteria in Solid Tumors; RECIST, Response Evaluation Criteria in Solid Tumors; NM, Not Mentioned; RT-PCR, Reverse Transcription Polymerase Chain Reaction; RNA, Ribonucleic Acid; miR, MicroRNA. ↓: decreased expression ↑: increased expression.

### 3.3 Quality assessment

**Figure 2:**
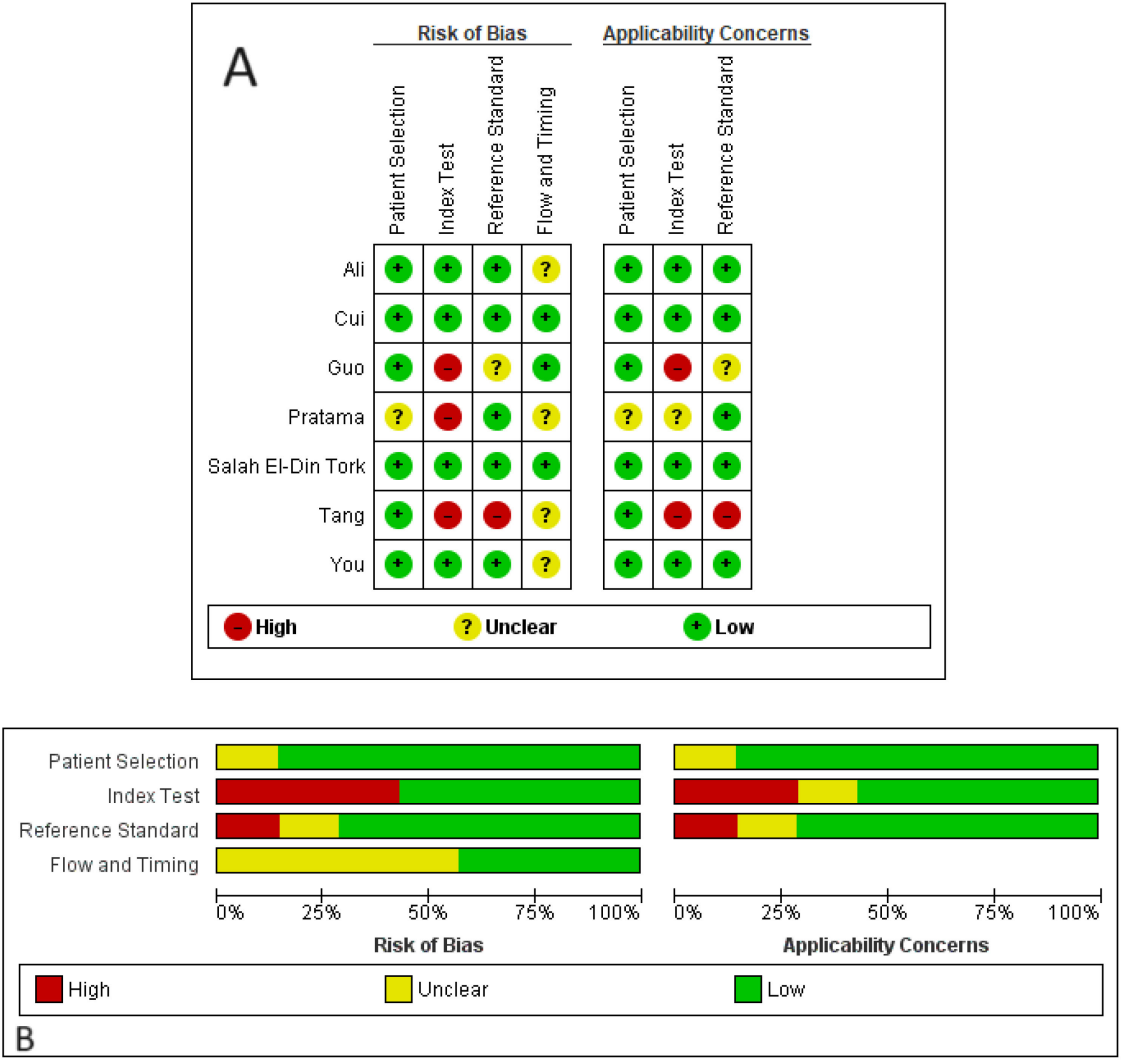
Risks of bias and applicability concerns of the included studies based on the QUADAS-2 tool. **(A)** quality assessment for each study; **(B)** quality assessment for each domain.

The quality of the studies was evaluated using the QUADAS-2 tool (**Figure 2**). Regarding the risk of bias, there was an overall low risk in the patient selection domain, with only one study (Pratama et al.) showing unclear risk due to not specifying clear patient selection criteria. In the index test domain, several studies included patients with partial response, stable disease, or relapsed cases in non-responder groups, leading to high risks of bias. Similarly, in the reference standard domain, some high and unclear risks of bias were detected due to not mentioning the response evaluation method (e.g., RECIST or mRECIST). In the flow and timing domain, four out of seven studies did not mention the interval between the index test and the reference standard, resulting in some unclear risks of bias. Regarding applicability concerns, only the index test showed moderate to high applicability concerns due to including patients with partial response, stable disease, or relapsed cases in non-responder groups.

### 3.4 Diagnostic performance of studies

**Figure 3:**
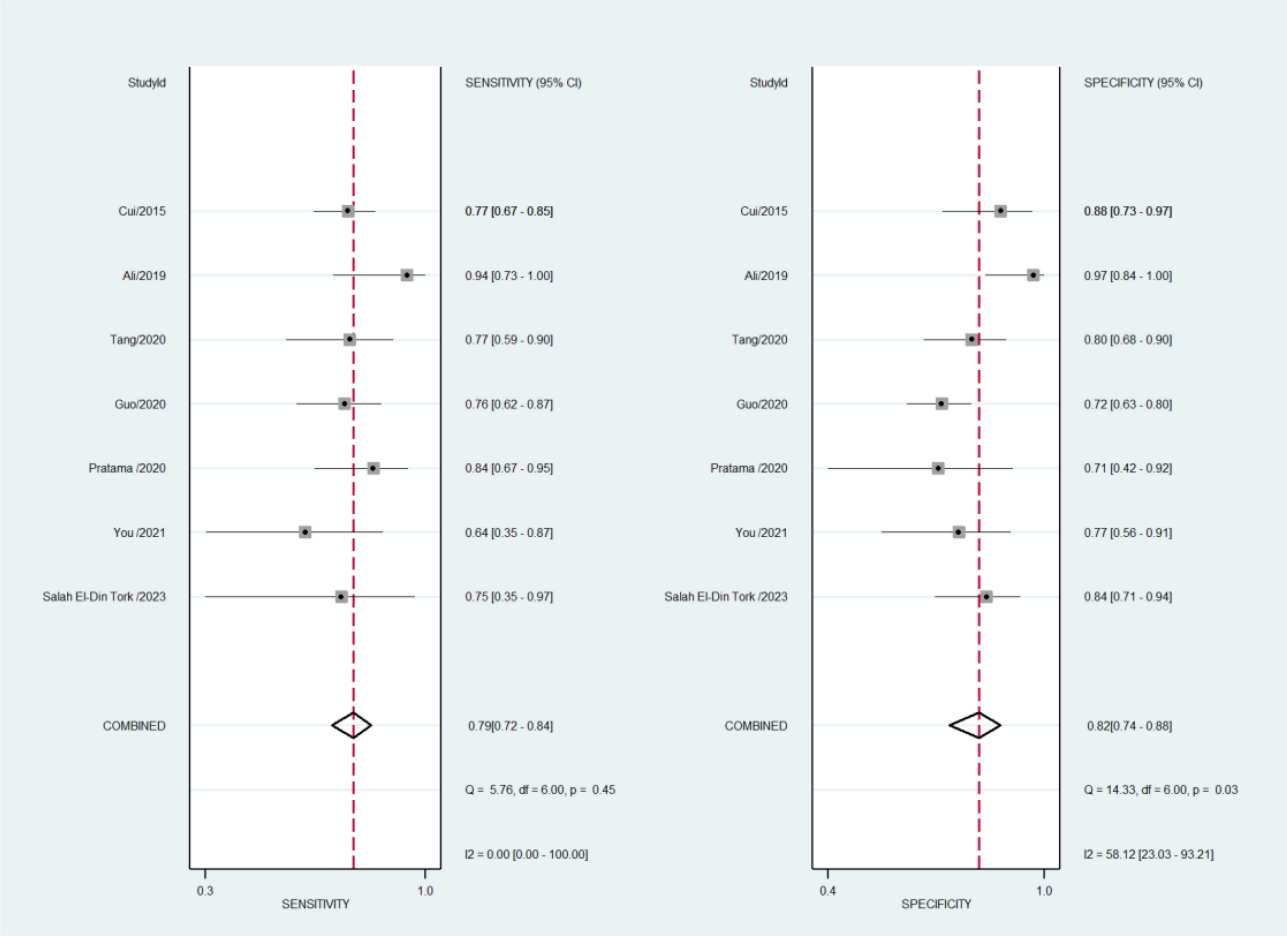
Coupled forest plots for sensitivity and specificity of miRNAs for evaluating response to TACE.

**Figure 4:**
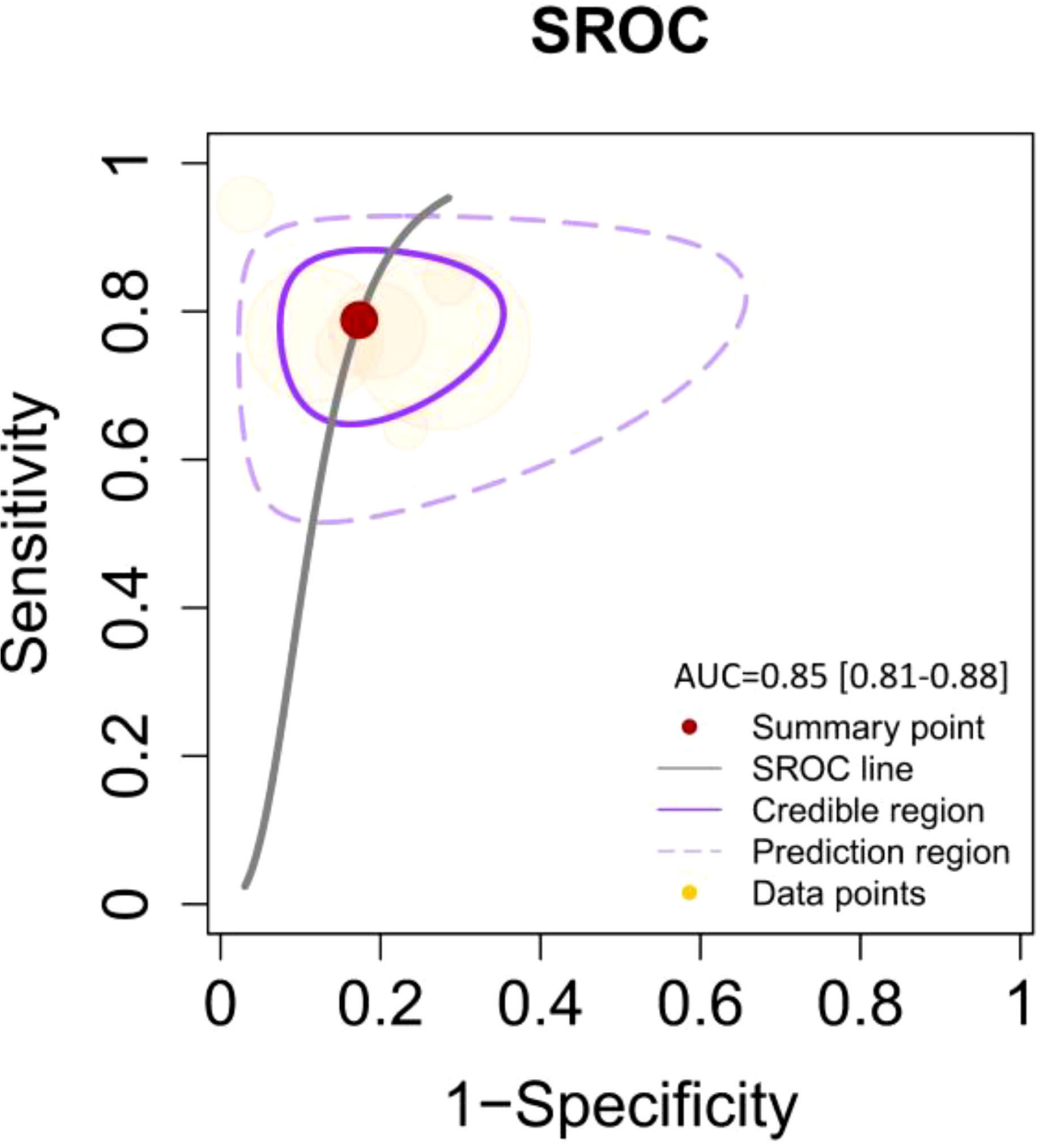
SROC curve illustrating the diagnostic performance with an AUC of 0.85 [95% CI: 0.81–0.88]. The curve includes the summary point (red dot), SROC line (solid black line), credible region (purple shaded area), prediction region (dashed purple line), and data points (yellow dots) representing individual studies.

By including the diagnostic performance of 7 miRNAs in a bivariate model, the pooled sensitivity (SENS), specificity (SPEC), PLR, NLR, and DOR were 0.79 [0.72, 0.84], 0.82 [0.74, 0.88], 4.5 [3.0, 6.7], 0.26 [0.19, 0.35], and 17 (9, 33), respectively. The coupled forest plot for sensitivity and specificity is depicted in **Figure 3**. Additionally, the pooled AUC derived from the SROC curve was 0.85 [0.81, 0.88], demonstrating excellent diagnostic accuracy (**Figure 4**).

### 3.5 Inter-study heterogeneity evaluation

We observed no significant heterogeneity in the pooled sensitivity (I^2^ = 0; p=0.45 for Cochran’s Q test). In contrast, the pooled specificity showed moderate heterogeneity (I^2^ = 58.12; p=0.03). To rule out the threshold effect as a potential source of inter-study heterogeneity, we employed MetaDiSc software to calculate the Spearman correlation coefficient, which resulted in r=-0.214 (p=0.645), confirming that the threshold effect was not responsible. Consequently, we conducted a meta-regression analysis using STATA software to identify the sources of heterogeneity. Our findings revealed that using RECIST criteria instead of mRECIST for treatment response evaluation and studies conducted in Egypt (which also used miRNeasy kit for RNA extraction) significantly contributed to the observed inter-study heterogeneity (Joint model analysis in **Table 2**).

**Table 2:** Subgroup analysis and heterogeneity exploration.

Variables		N	Sensitivity	P_1_	Specificity	P_2_	Joint model analysis		
							I^2^	LRT chi^2^	P value
**Sample size**	<50	2	0.78 [0.66 - 0.91]	0.08	0.76 [0.58 - 0.93]	0.69	0	0.91	0.63
	≥51	5	0.79 [0.72 - 0.85]		0.84 [0.76 - 0.91]				
**Response Group**	CR+PR+SD vs. Relapse	2	0.77 [0.67 - 0.86]	0.00	0.75 [0.68 - 0.82]	0.00	30	2.85	0.24
	Other	5	0.79 [0.73 - 0.85]		0.86 [0.80 - 0.91]				
**Response Group**	CR+PR vs. NR	4	0.78 [0.71 - 0.85]	0.00	0.87 [0.81 - 0.93]	0.10	57	4.65	0.10
	Other	3	0.79 [0.71 - 0.86]		0.75 [0.68 - 0.81]				
**Specimen**	Serum	6	0.79 [0.73 - 0.84]	0.99	0.82 [0.74 - 0.89]	0.22	0	0.20	0.91
	Plasma	1	0.75 [0.45 - 1.00]		0.85 [0.70 - 0.99]				
**Extraction Method**	miRNeasy kit	2	0.88 [0.76 - 1.00]	0.90	0.90 [0.83 - 0.97]	0.53	66	5.93	**0.05**
	Other	5	0.77 [0.71 - 0.83]		0.77 [0.71 - 0.84]				
**Response Evaluation Method**	mRECIST	4	0.77 [0.69 - 0.86]	0.00	0.76 [0.69 - 0.84]	0.00	46	3.73	0.15
	Other	3	0.79 [0.72 - 0.87]		0.87 [0.81 - 0.94]				
**Response Evaluation Method**	RECIST	2	0.80 [0.72 - 0.87]	0.01	0.93 [0.86 - 0.99]	0.94	72	7.17	**0.03**
	Other	5	0.77 [0.70 - 0.84]		0.77 [0.71 - 0.82]				
**TACE efficacy interval window**	3 Months	2	0.77 [0.65 - 0.89]	0.03	0.77 [0.67 - 0.88]	0.00	0	1.14	0.56
	Other	5	0.79 [0.73 - 0.85]		0.84 [0.78 - 0.91]				
**Country**	Egypt	2	0.88 [0.76 - 1.00]	0.90	0.90 [0.83 - 0.97]	0.53	66	5.93	**0.05**
	Other	5	0.77 [0.71 - 0.83]		0.77 [0.71 - 0.84]				
**Country**	China	4	0.76 [0.69 - 0.82]	0.00	0.78 [0.71 - 0.86]	0.00	56	1	100
	Other	3	0.86 [0.77 - 0.95]		0.87 [0.79 - 0.95]				

N, Number of studies or samples included in the analysis; P1, P-value for statistical significance of sensitivity difference; P2, P-value for statistical significance of specificity difference; I2, I-squared statistic, measuring the percentage of variability in results due to heterogeneity rather than chance; LRT, Likelihood Ratio Test, a statistical test used to compare the goodness of fit of two models; Chi2, Chi-squared statistic, a measure used in hypothesis testing to evaluate the differences between observed and expected data. Bold numbers: statistically significant.

### 3.6 Subgroup meta-analysis

The following analysis provides insights into the sensitivity and specificity of miRNA diagnostic performance across various subgroups, with statistical comparisons between groups using p1 and p2 values where significant.

#### 3.6.1 Sample size

Studies with sample sizes less than 50 (N=2) had a sensitivity of 0.78 [0.66 - 0.91] (P1 = 0.08) and a specificity of 0.76 [0.58 - 0.93] (P2 = 0.69). There was no significant heterogeneity (I2 = 0, LRT chi2 = 0.91, P=0.63). Studies with sample sizes of 51 or more (N=5) reported a sensitivity of 0.79 [0.72 - 0.85] and a specificity of 0.84 [0.76 - 0.91]. The p-values suggest no significant difference between the sensitivity and specificity of the two groups.

#### 3.6.2 miRNA isolation method

Studies that used miRNeasy kit (N=2) for RNA extraction had a sensitivity of 0.88 [0.76 - 1.00] (P1 = 0.90) and specificity of 0.90 [0.83 - 0.97] (P2 = 0.53), with moderate heterogeneity (I2 = 66, LRT chi2 = 5.93, P=0.05). Studies that used other tools (N=5) showed a sensitivity of 0.77 [0.71 - 0.83] and a specificity of 0.77 [0.71 - 0.84]. The P-values suggest no significant difference between the sensitivity and specificity of these groups.

#### 3.6.3 Response group

##### 3.6.3.1 CR+PR+SD vs. relapse

For CR+PR+SD vs. relapse (N=2), the sensitivity was 0.77 [0.67 - 0.86] (P1 = 0.00) and specificity was 0.75 [0.68 - 0.82] (P2 = 0.00), with moderate heterogeneity (I2 = 30, LRT chi2 = 2.85, P=0.24). Other response groups (N=5) had a sensitivity of 0.79 [0.73 - 0.85] and specificity of 0.86 [0.80 - 0.91]. The significant p-values indicate notable differences in sensitivity and specificity between these groups.

##### 3.6.3.2 CR+PR vs. NR

For CR+PR vs. NR (N=4), sensitivity was 0.78 [0.71 - 0.85] (P1 = 0.00) and specificity was 0.87 [0.81 - 0.93] (P2 = 0.10), showing significant heterogeneity (I2 = 57, LRT chi2 = 4.65, P=0.10). Other response groups (N=3) showed a sensitivity of 0.79 [0.71 - 0.86] and specificity of 0.75 [0.68 - 0.81]. Here, P1 indicates a significant difference in sensitivity.

#### 3.6.4 Specimen source

Studies using serum samples (N=6) had a sensitivity of 0.79 [0.73 - 0.84] (P1 = 0.99) and specificity of 0.82 [0.74 - 0.89] (P2 = 0.22), with no significant heterogeneity (I2 = 0, LRT chi2 = 0.20, P=0.91). One study using plasma samples reported a sensitivity of 0.75 [0.45 - 1.00] and specificity of 0.85 [0.70 - 0.99]. The P-values suggest no significant difference due to the limited number of plasma studies.

#### 3.6.5 mRECIST response evaluation method

Studies using mRECIST (N=4) showed a sensitivity of 0.77 [0.69- 0.86] (P1 = 0.00) and specificity of 0.76 [0.69 - 0.84] (P2 = 0.00), with moderate heterogeneity (I2 = 46, LRT chi2 = 3.73, P=0.15). Studies using other methods (N=3) reported a sensitivity of 0.79 [0.72 - 0.87] and specificity of 0.87 [0.81 - 0.94]. The significant p-values indicate notable differences in sensitivity and specificity.

#### 3.6.6 RECIST response evaluation method

Studies using RECIST (N=2) showed a sensitivity of 0.80 [0.72 - 0.87] (P1 = 0.01) and specificity of 0.93 [0.86 - 0.99] (P2 = 0.94), with significant heterogeneity (I2 = 72, LRT chi2 = 7.17, P=0.03). Studies using other evaluation methods (N=5) showed a sensitivity of 0.77 [0.70 - 0.84] and specificity of 0.77 [0.71 - 0.82]. P1 indicates a significant difference in sensitivity.

#### 3.6.7 TACE efficacy interval window

Studies with a 3-month interval (N=2) reported a sensitivity of 0.77 [0.65 - 0.89] (P1 = 0.03) and specificity of 0.77 [0.67 - 0.88] (P2 = 0.00), with no significant heterogeneity (I2 = 0, LRT chi2 = 1.14, P=0.56). Studies with other intervals (N=5) reported a sensitivity of 0.79 [0.73 - 0.85] and specificity of 0.84 [0.78 - 0.91]. The significant p-values indicate differences in both sensitivity and specificity.

#### 3.6.8 Country

##### 3.6.8.1 Egypt

Studies conducted in Egypt (N=2) had a sensitivity of 0.88 [0.76 - 1.00] (P1 = 0.90) and specificity of 0.90 [0.83 - 0.97] (P2 = 0.53), with moderate heterogeneity (I2 = 66, LRT chi2 = 5.93, P=0.05). Studies from other countries (N=5) showed a sensitivity of 0.77 [0.71 - 0.83] and specificity of 0.77 [0.71 - 0.84]. The P-values suggest no significant difference between the sensitivity and specificity of these groups.

##### 3.6.8.2 China

Studies conducted in China (N=4) showed a sensitivity of 0.76 [0.69 - 0.82] (P1 = 0.00) and specificity of 0.78 [0.71 - 0.86] (P2 = 0.00), with significant heterogeneity (I2 = 56, LRT chi2 = 1.00, P=0.00). Studies from other countries (N=3) had a sensitivity of 0.86 [0.77 - 0.95] and specificity of 0.87 [0.79 - 0.95]. The significant p-values indicate notable differences in sensitivity and specificity.

### 3.7 Publication bias

**Figure 5:**
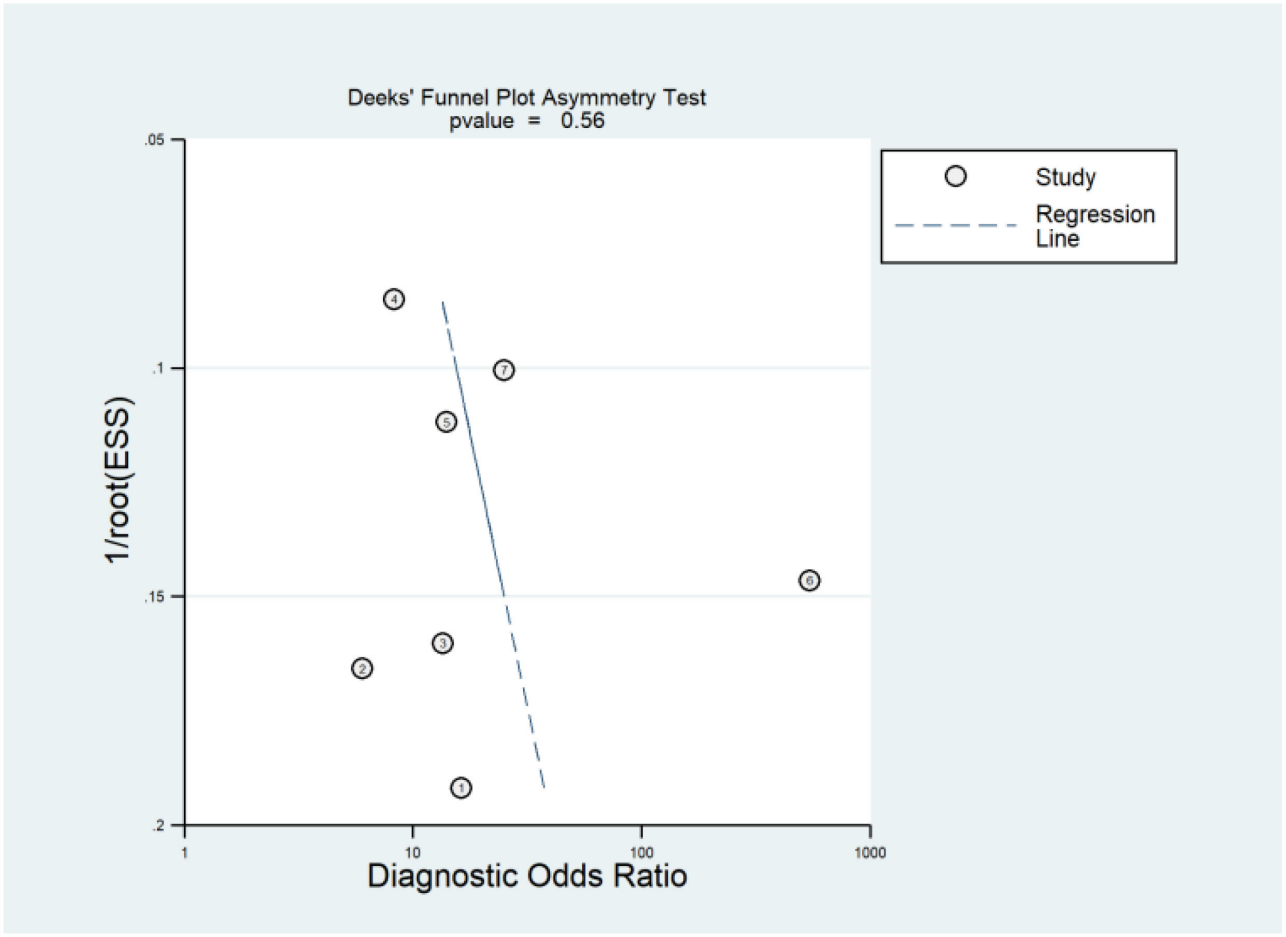
Deeks’ funnel plot is a graphical method used to assess publication bias in meta-analyses of diagnostic accuracy studies. Deeks’ asymmetry test complements the funnel plot by providing a statistical measure to detect such bias, where a p-value greater than 0.05 generally indicates no significant publication bias.

Publication bias occurs when the results of research studies influence their likelihood of being published. Typically, studies with positive or significant results are more likely to be published than those with negative or null findings. This bias can distort the overall understanding of a research topic because the available literature is not fully representative of all conducted studies. Deeks’ funnel plot is a graphical tool used to detect publication bias in meta-analyses of diagnostic test accuracy studies. It plots the inverse of the square root of the effective sample size against the DOR for each study. In the absence of publication bias, the plot resembles a symmetrical inverted funnel. An asymmetrical funnel suggests the presence of publication bias, where smaller studies with non-significant or less favorable results are underrepresented. Deeks’ asymmetry test, often conducted alongside the funnel plot, provides a statistical measure to confirm the presence of publication bias. The Deeks’ asymmetry test yielded a p-value of 0.55, indicating that there is no statistically significant evidence of publication bias. Furthermore, the funnel plot (**Figure 5**) appeared symmetrical, reinforcing the conclusion that publication bias is likely not a significant issue in the dataset under analysis.

### 3.8 Clinical diagnostic value

**Figure 6:**
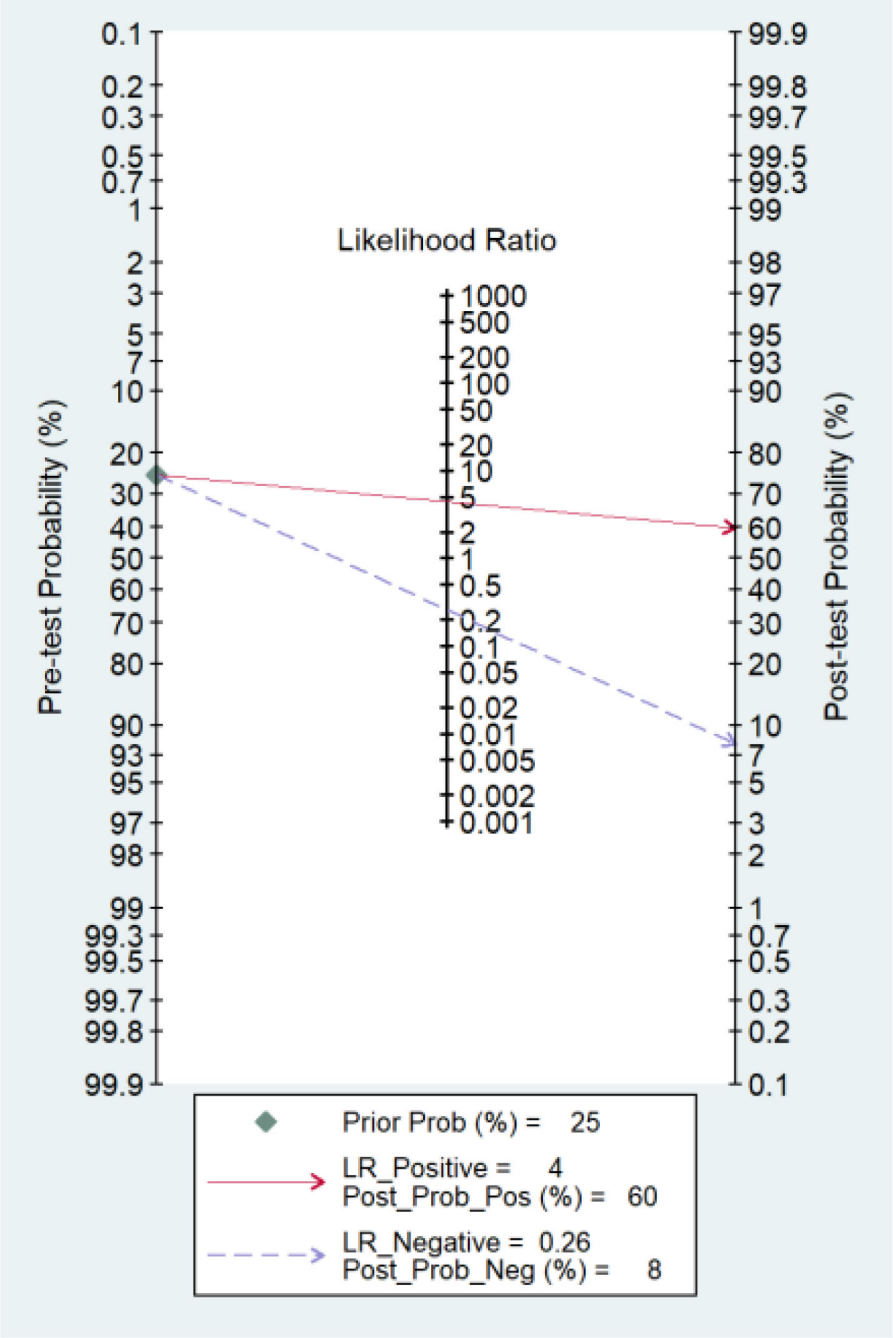
Fagan’s nomogram showing the clinical utility of miRNAs in response to TACE in HCC. Prior Prob (%), Prior Probability; LR_Positive, Likelihood Ratio Positive; Post_Prob_Pos (%), Post-test Probability Positive; LR_Negative, Likelihood Ratio Negative; Post_Prob_Neg (%), Post-test Probability Negative.

Fagan’s nomogram is a tool used to interpret diagnostic test results by combining pretest probability with likelihood ratios to estimate post-test probability. In this analysis, the use of miRNAs as a diagnostic tool for predicting response to TACE shows significant potential. If the pretest probability (the likelihood of having the condition before the test) is 25%, a positive test result increases the probability of the condition to 60%, with a PLR of 4. This means that a positive miRNA test makes it four times more likely that the patient has the condition compared to before the test. Conversely, if the pretest probability is the same 25%, a negative test result lowers the probability of having the condition to 8%, with a NLR of 0.26. This indicates that a negative miRNA test result significantly reduces the likelihood of the patient having the condition. Thus, miRNAs appear to be a valuable diagnostic tool, substantially altering the probability of disease presence based on test outcomes (**Figure 6**).

### 3.9 Sensitivity analysis

**Figure 7:**
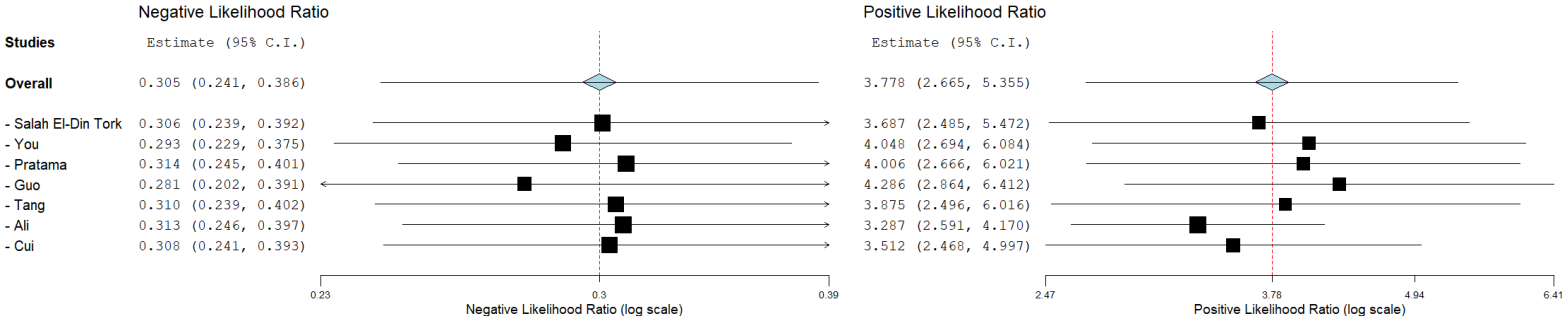
Sensitivity analysis using a leave-one-out approach for pooled estimates of sensitivity, specificity, and DOR. The top panel displays the pooled sensitivity estimates (0.774; 95% CI: 0.716-0.823) with the contribution of individual studies highlighted. The middle panel shows the pooled specificity estimates (0.803; 95% CI: 0.729-0.861). The bottom panel represents the pooled DOR estimates (14.593; 95% CI: 7.554-28.188). The minimal changes in effect size illustrate the stability of the pooled estimates after the sequential exclusion of individual studies.

The leave-one-out sensitivity analysis reveals minimal changes in the pooled effect sizes when individual studies are excluded (**Figure 7**). Sensitivity ranged from 0.765 to 0.784, specificity varied from 0.779 to 0.826, and the DOR ranged from 11.658 to 17.635. These ranges indicate that the pooled estimates are robust, as the observed variations remain within overlapping confidence intervals and do not substantially alter the overall results. This demonstrates that the findings of the meta-analysis are reliable and not overly influenced by any single study.

## 4 Discussion

TACE is a pivotal treatment for HCC, especially in patients with intermediate-stage disease who are ineligible for surgical removal or liver transplants. TACE functions by combining avascular necrosis and localized chemotherapy, which is administered directly through the hepatic artery that supplies blood to the tumor. This method effectively cuts off the tumor’s blood supply, causing tumor cell death, while simultaneously delivering high doses of chemotherapy to the cancerous area. TACE has demonstrated significant improvements in survival rates and symptom management, without substantially impairing liver function, assuming the patient’s liver function is reasonably preserved and there are no issues such as portal vein thrombosis or ascites. This procedure can be used alone or (19, 20) in conjunction with other treatments like radiation therapy, radiofrequency ablation (RFA), and systemic therapies like sorafenib for more advanced cases. The effectiveness of TACE hinges on careful patient selection and customized treatment plans to ensure the best outcomes and minimize potential risks (20).

Evaluating the response to TACE treatment is vital because it provides critical information on the effectiveness of the therapy in targeting liver tumors. Accurate assessment allows healthcare professionals to determine whether the treatment is successfully reducing tumor size, necrosis, and viability, which directly correlates with patient prognosis and survival rates. Moreover, evaluating treatment response aids in making informed decisions about subsequent therapeutic strategies, such as additional TACE sessions, alternative treatments, or supportive care. It also helps in identifying potential complications early, ensuring timely interventions to mitigate adverse effects and improve overall patient outcomes (19, 20).

To the best of our knowledge, this study is the first to evaluate the diagnostic performance of serum/plasma-derived biomarkers in predicting response to TACE therapy in HCC patients. Typically, therapeutic response in HCC is evaluated using imaging methods such as CT scans and MRI. While these imaging techniques are standard, the advent of artificial intelligence-based models (radiomics) has led to the development of models capable of predicting response to TACE in HCC cases, achieving an impressive diagnostic performance with an AUC of 0.93 (21).

However, these methods have significant limitations. The cost of advanced imaging and the infrastructure required for AI-based analysis can be prohibitively high, limiting accessibility in resource-limited settings. The accuracy of radiomics models is highly dependent on the quality of the imaging data, which can vary significantly across different machines and institutions. There is considerable variability in the interpretation of imaging results, leading to inconsistent outcomes. Radiomics models also require large, well-annotated datasets for training, which may not be available for all patient populations. Additionally, issues with reproducibility and generalizability of these models across diverse clinical settings pose further challenges (22–24).

This study, therefore, fills a crucial gap by exploring alternative, potentially more accessible, and less resource-intensive biomarkers that could complement or enhance current imaging-based diagnostic strategies. By evaluating serum/plasma-derived biomarkers, we aim to provide a more universally applicable method for predicting response to TACE therapy in HCC, which could lead to improved patient outcomes and more personalized treatment approaches.

The findings from this meta-analysis underscore the significant potential of miRNAs as predictive biomarkers for assessing the response to TACE in HCC patients. The pooled sensitivity and specificity of 0.79 and 0.82, respectively, along with an AUC of 0.85, demonstrate that miRNAs possess excellent diagnostic accuracy. These results indicate that miRNAs could serve as reliable indicators for predicting which patients are likely to respond favorably to TACE, thereby enhancing the ability to personalize treatment strategies and improve patient outcomes.

One of the key strengths of this study is the comprehensive analysis of miRNA diagnostic performance across multiple studies, encompassing diverse geographical locations and varied patient populations. The inclusion of studies from different countries, such as Egypt, China, and Italy, highlights the broad interest and applicability of miRNA research in HCC. This geographical diversity also reinforces the robustness of the findings, suggesting that miRNAs could be universally effective biomarkers, irrespective of population-specific genetic and environmental factors.

Among the analyzed microRNAs, miR-210 and miR-373 demonstrated elevated expression, functioning as oncogenes in HCC patients. Notably, their expression significantly diminished after undergoing TACE therapy. Conversely, certain other microRNAs displayed a reverse pattern of expression: MiR-373 acts as an oncogenic miRNA in hepatocellular carcinoma by promoting cell proliferation and the G1/S cell cycle transition through downregulation of the tumor suppressor PPP6C (25). Similarly, miR-210 promotes HCC progression by enhancing autophagy in M2-polarized macrophages through inhibition of the PI3K/AKT/mTOR signaling pathway, thereby fostering tumor cell proliferation, invasion, and immune evasion in the tumor microenvironment (26). In addition, miR-210 is a specific biomarker for differentiating HCC from other metastatic lesions in the liver (27). Therefore, their downregulation following TACE treatment might be an indicator of the treatment efficacy (12, 15). In contrast, substantial evidence highlights the tumor-suppressive role of miR-1271 in HCC, demonstrating its involvement in inhibiting cell proliferation (28), enhancing radiosensitivity (29), and suppressing epithelial-to-mesenchymal transition (30). Similarly, miR-214 is downregulated in HCC patients, and restoring its expression has been shown to inhibit cell proliferation (31), migration (32), metabolism (32), angiogenesis (33), and the expression of β-catenin protein (34), a key factor in HCC progression (35). In addition, miR-133b is downregulated in HCC patients (36), leading to reduced growth factor levels in connective tissues (37). Its expression suppresses cell proliferation (36), migration (38), and invasion (38), is positively associated with better patient prognosis (39), and enhances sensitivity to cisplatin treatment (40). More importantly, researchers have discovered that encapsulating miR-335 in exosomes enhances its stability, bioavailability, and therapeutic efficacy, overcoming resistance mechanisms in HCC (41).

The subgroup analyses provided further insights into factors affecting the diagnostic performance of miRNAs. Significant differences were observed in diagnostic accuracy based on response criteria and geographical location. For instance, studies using the mRECIST criteria showed moderate heterogeneity, while those using RECIST criteria exhibited higher diagnostic accuracy. This suggests that standardized response evaluation methods are crucial for accurately assessing the effectiveness of miRNAs as predictive biomarkers. Additionally, the higher diagnostic performance observed in studies from Egypt suggests potential regional differences in miRNA expression or detection methodologies that warrant further investigation.

Differences in RNA extraction methods and endogenous controls can introduce variability in the results and cause heterogeneity. This is particularly relevant in the context of small, non-coding RNA molecules like miRNAs, where consistency in methodology is crucial for reliable quantification. The included studies employed various RNA extraction methods. While methodological differences exist, the extraction methods used across the studies are validated techniques widely accepted in the field. Additionally, studies were included based on stringent criteria that ensured reliable miRNA quantification and diagnostic reporting. Furthermore, the extraction methods varied among the studies, with only two utilizing the miRNeasy kit. We conducted a new subgroup analysis focusing on these two studies to address this. Our findings indicate that RNA extraction methods might contribute to interstudy heterogeneity. However, since both studies were conducted in Egypt, reaching a definitive conclusion remains challenging. Lastly, we performed a sensitivity analysis to evaluate the robustness of the results. We observed that if one study is removed one by one (leave one out analysis), the overall pooled results for sensitivity, specificity, and DOR do not change significantly. These findings suggest that miRNAs demonstrate promising diagnostic potential in predicting the response to TACE in HCC patients. However, further validation with larger and more diverse patient cohorts is essential before their incorporation into routine clinical practice.

The clinical implications of these findings are substantial. Incorporating miRNA profiling into routine clinical practice could enhance the stratification of HCC patients, allowing clinicians to identify those who are more likely to benefit from TACE. This targeted approach could lead to better treatment outcomes, reduced side effects, and more efficient use of healthcare resources. Furthermore, understanding the limitations and sources of variability in current miRNA research could guide future studies towards more standardized and reliable approaches, ultimately improving the reliability of miRNAs as predictive biomarkers.

## 5 Limitations and future perspectives

### 5.1 Limitations

This study has several limitations. First, the small number of included studies and limited sample sizes may restrict the generalizability of the findings. Second, heterogeneity was observed across studies, partly due to differences in RNA extraction methods, miRNA quantification techniques, and response evaluation criteria (e.g., RECIST vs. mRECIST). Third, most studies were conducted in specific geographic regions, such as Egypt and China, which may limit the applicability of the results to broader populations. Finally, the absence of standardized methodologies for miRNA profiling introduces variability, impacting the robustness of diagnostic performance metrics.

### 5.2 Future perspectives

To address these limitations and advance the field, future research should focus on the following:

- Standardization of Methodologies: Develop and adopt standardized protocols for RNA extraction, miRNA quantification, and normalization using consistent endogenous controls.
- Uniform Response Evaluation: Establish consensus on response evaluation criteria (e.g., mRECIST or RECIST) to ensure consistency in defining treatment outcomes.
- Larger, Multicenter Studies: Conduct prospective, multicenter studies with diverse patient populations to validate findings and improve generalizability.
- Integration with Advanced Technologies: Explore combining miRNA profiling with radiomics or machine learning-based models to enhance diagnostic accuracy and predictive power.
- Clinical Translation: Evaluate the cost-effectiveness, feasibility, and real-world application of miRNA biomarkers in clinical settings to facilitate their incorporation into routine practice.

## 6 Conclusion

In conclusion, this meta-analysis provides compelling evidence for the diagnostic potential of miRNAs in predicting the response to TACE in HCC patients. The high sensitivity and specificity, along with the robust AUC, highlight the promise of miRNAs as valuable predictive biomarkers. Implementing miRNA profiling in clinical practice could revolutionize the management of HCC, leading to more personalized and effective treatment strategies. Further research is needed to address the existing limitations and enhance the reliability of miRNAs as predictive tools in HCC therapy.

## Data Availability

The original contributions presented in the study are included in the article/supplementary material. Further inquiries can be directed to the corresponding author.

## References

[1] Huang J Lok V Ngai CH Chu C Patel HK Thoguluva Chandraseka V . Disease burden, risk factors, and recent trends of liver cancer: a global country-level analysis. Liver Cancer. (2021) 10:330–45. doi: 10.1159/000515304 PMC8339459 34414121

[2] Wang W Wei C . Advances in the early diagnosis of hepatocellular carcinoma. Genes Dis. (2020) 7:308–19. doi: 10.1016/j.gendis.2020.01.014 PMC7452544 32884985

[3] Ghanaati H Mohammadifard M Mohammadifard M . A review of applying transarterial chemoembolization (TACE) method for management of hepatocellular carcinoma. J Fam Med Prim Care. (2021) 10:3553–60. doi: 10.4103/jfmpc.jfmpc_2347_20 PMC8653440 34934646

[4] Fite EL Makary MS . Transarterial chemoembolization treatment paradigms for hepatocellular carcinoma. Cancers (Basel). (2024) 16:2430. doi: 10.3390/cancers16132430 39001491 PMC11240648

[5] Pinto E Pelizzaro F Cardin R Battistel M Palano G Bertellini F . HIF-1α and VEGF as prognostic biomarkers in hepatocellular carcinoma patients treated with transarterial chemoembolization. Dig Liver Dis. (2024) 56:872–9. doi: 10.1016/j.dld.2023.09.019 37783655

[6] Johnson P Zhou Q Dao DY Lo YMD . Circulating biomarkers in the diagnosis and management of hepatocellular carcinoma. Nat Rev Gastroenterol Hepatol. (2022) 19:670–81. doi: 10.1038/s41575-022-00620-y 35676420

[7] D’Abundo L Bassi C Callegari E Moshiri F Guerriero P Michilli A . Circulating microRNAs as biomarkers for stratifying different phases of liver cancer progression and response to therapy. Sci Rep. (2024) 14:18551. doi: 10.1038/s41598-024-69548-4 39122875 PMC11315904

[8] Afshari A Yaghobi R Karimi MH Mowla J . Alterations in MicroRNA gene expression profile in liver transplant patients with hepatocellular carcinoma. BMC Gastroenterol. (2021) 21:262. doi: 10.1186/s12876-020-01596-2 34118888 PMC8199419

[9] Morishita A Oura K Tadokoro T Fujita K Tani J Masaki T . MicroRNAs in the pathogenesis of hepatocellular carcinoma: A review. Cancers (Basel). (2021) 13:514. doi: 10.3390/cancers13030514 33572780 PMC7866004

[10] Pratama MY Visintin A Crocè LS Tiribelli C Pascut D . Circulatory miRNA as a biomarker for therapy response and disease-free survival in hepatocellular carcinoma. Cancers. (2020) 12. doi: 10.3390/cancers12102810 PMC7601056 33003646

[11] Guo Z Wang J Li L Liu R Fang J Tie B . Value of miR-1271 and glypican-3 in evaluating the prognosis of patients with hepatocellular carcinoma after transcatheter arterial chemoembolization. World J Clin Cases. (2020) 8:3493. doi: 10.12998/wjcc.v8.i16.3493 32913856 PMC7457095

[12] Tork ASE-D Kamel AAF Zaki MA Abo El-Wafa RAH El-Assar OS Ibrahim Abdelkarem OA . Circulating miRNA-373 as a predictor of response to super-selective transarterial chemoembolization bridging therapy in hepatocellular carcinoma patients awaiting liver transplantation. Asian Pacific J Cancer Prev. (2023) 24:291–9. doi: 10.31557/APJCP.2023.24.1.291 PMC10152864 36708579

[13] Liu M Liu J Wang L Wu H Zhou C Zhu H . Association of serum microRNA expression in hepatocellular carcinomas treated with transarterial chemoembolization and patient survival. PLoS One. (2014) 9:e109347. doi: 10.1371/journal.pone.0109347 25275448 PMC4183700

[14] Salameh J-P Bossuyt PM McGrath TA Thombs BD Hyde CJ Macaskill P . Preferred reporting items for systematic review and meta-analysis of diagnostic test accuracy studies (PRISMA-DTA): explanation, elaboration, and checklist. bmj. (2020) 370:388–96. doi: 10.1136/bmj.m2632 32816740

[15] You R Jiang H Xu QY Yin GW . Preintervention MCP-1 serum levels as an early predictive marker of tumor response in patients with hepatocellular carcinoma undergoing transarterial chemoembolization. Transl Cancer Res. (2021) 10:966–76. doi: 10.21037/tcr-20-2791 PMC8797576 35116424

[16] Tang L Zhang XM . Serum TSGF and miR-214 levels in patients with hepatocellular carcinoma and their predictive value for the curative effect of transcatheter arterial chemoembolization. Ann Palliat Med. (2020) 9:2111–7. doi: 10.21037/apm-20-1224 32762227

[17] Ali HEA Emam AA Zeeneldin AA Srour R Tabashy R El-Desouky ED . Circulating miR-26a, miR-106b, miR-107 and miR-133b stratify hepatocellular carcinoma patients according to their response to transarterial chemoembolization. Clin Biochem. (2019) 65:45–52. doi: 10.1016/j.clinbiochem.2019.01.002 30653948 PMC6397777

[18] Cui LM Hu Y Bai B Zhang SD . Serum miR-335 level is associated with the treatment response to trans-arterial chemoembolization and prognosis in patients with hepatocellular carcinoma. Cell Physiol Biochem. (2015) 37:276–83. doi: 10.1159/000430352 26305026

[19] Voizard N Cerny M Assad A Billiard J-S Olivié D Perreault P . Assessment of hepatocellular carcinoma treatment response with LI-RADS: a pictorial review. Insights Imaging. (2019) 10:1–22. doi: 10.1186/s13244-019-0801-z 31853668 PMC6920285

[20] Kotsifa E Vergadis C Vailas M Machairas N Kykalos S Damaskos C . Transarterial chemoembolization for hepatocellular carcinoma: why, when, how? J Pers Med. (2022) 12:436. doi: 10.1055/s-0033-1333648 35330436 PMC8955120

[21] Feng L Chen Q Huang L Long L . Radiomics features of computed tomography and magnetic resonance imaging for predicting response to transarterial chemoembolization in hepatocellular carcinoma: a meta-analysis. Front Oncol. (2023) 13:1194200. doi: 10.3389/fonc.2023.1194200 37519801 PMC10374837

[22] Cobo M Menéndez Fernández-Miranda P Bastarrika G Lloret Iglesias L . Enhancing radiomics and Deep Learning systems through the standardization of medical imaging workflows. Sci data. (2023) 10:732. doi: 10.1038/s41597-023-02641-x 37865635 PMC10590396

[23] Jha AK Mithun S Sherkhane UB Dwivedi P Puts S Osong B . Emerging role of quantitative imaging (radiomics) and artificial intelligence in precision oncology. Explor Target Anti-tumor Ther. (2023) 4:569. doi: 10.37349/etat PMC10501896 37720353

[24] Horvat N Papanikolaou N Koh D-M . Radiomics beyond the hype: a critical evaluation toward oncologic clinical use. Radiol Artif Intell. (2024) 6:e230437. doi: 10.1148/ryai.230437 38717290 PMC11294952

[25] Wu N Liu X Xu X Fan X Liu M Li X . MicroRNA-373, a new regulator of protein phosphatase 6, functions as an oncogene in hepatocellular carcinoma. FEBS J. (2011) 278:2044–54. doi: 10.1111/j.1742-4658.2011.08120.x 21481188

[26] Bi S Zhang Y Zhou J Yao Y Wang J Fang M . miR-210 promotes hepatocellular carcinoma progression by modulating macrophage autophagy through PI3K/AKT/mTOR signaling. Biochem Biophys Res Commun. (2023) 662:47–57. doi: 10.1016/j.bbrc.2023.04.055 37099810 PMC11153875

[27] Ahmed EK Fahmy SA Effat H Wahab AHA . Circulating miR-210 and miR-1246 as potential biomarkers for differentiating hepatocellular carcinoma from metastatic tumors in the liver. J Med Biochem. (2019) 38:109. doi: 10.2478/jomb-2018-0010 30867638 PMC6411000

[28] Qin A Zhu J Liu X Zeng D Gu M Lv C . MicroRNA−1271 inhibits cellular proliferation of hepatocellular carcinoma. Oncol Lett. (2017) 14:6783–8. doi: 10.3892/ol.2017.7052 PMC5696716 29181102

[29] Liu H-M Tan H-Y Lin Y Xu B-N Zhao W-H Xie Y-A . MicroRNA-1271-5p inhibits cell proliferation and enhances radiosensitivity by targeting CDK1 in hepatocellular carcinoma. J Biochem. (2020) 167:513–24. doi: 10.1093/jb/mvz114 32275316

[30] Li C Jiang Y Miao R Qu K Zhang J Liu C . MicroRNA-1271 functions as a metastasis and epithelial-mesenchymal transition inhibitor in human HCC by targeting the PTP4A1/c-Src axis. Int J Oncol. (2018) 52:536–46. doi: 10.3892/ijo.2017.4224 29345291

[31] Chen C Tao Z Li Y Li J Xu Y . MicroRNA214 expression inhibits HCC cell proliferation through PTK2b/ Pyk2. Cell Mol Biol. (2022) 68:20–5. doi: 10.14715/cmb/2022.68.1.4 35809332

[32] Yu Q Zhou J Jian Y Xiu Z Xiang L Yang D . MicroRNA-214 suppresses cell proliferation and migration and cell metabolism by targeting PDK2 and PHF6 in hepatocellular carcinoma. Cell Biol Int. (2020) 44:117–26. doi: 10.1002/cbin.11207 31329335

[33] Yahya SMM Yahya SMM . The Effect of miR-98 and miR-214 on Apoptotic and Angiogenic Pathways in Hepatocellular Carcinoma HepG2 Cells. Indian J Clin Biochem. (2020) 35:353–8. doi: 10.1007/s12291-019-00824-1 PMC7326845 32647414

[34] Karimkhanloo H Mohammadi-Yeganeh S Hadavi R Koochaki A Paryan M . Potential role of miR-214 in β-catenin gene expression within hepatocellular carcinoma. Mol Biol Rep. (2020) 47:7429–37. doi: 10.1007/s11033-020-05798-5 32901357

[35] Xu C Xu Z Zhang Y Evert M Calvisi DF Chen X . [amp]]beta;-Catenin signaling in hepatocellular carcinoma. J Clin Invest. (2022) 132. doi: 10.1172/jci154515 PMC8843739 35166233

[36] Luo Z Fan Y Liu X Liu S Kong X Ding Z . MiR-188-3p and miR-133b suppress cell proliferation in human hepatocellular carcinoma via post-transcriptional suppression of NDRG1. Technol Cancer Res Treat. (2021) 20:15330338211033074. doi: 10.1177/15330338211033074 34355586 PMC8358491

[37] Gjymishka A Pi L Oh S-H Jorgensen M Liu C Protopapadakis Y . miR-133b regulation of connective tissue growth factor: a novel mechanism in liver pathology. Am J Pathol. (2016) 186:1092–102. doi: 10.1016/j.ajpath.2015.12.022 PMC4861761 26945106

[38] Li H Xiang Z Liu Y Xu B Tang J . MicroRNA-133b inhibits proliferation, cellular migration, and invasion via targeting LASP1 in hepatocarcinoma cells. Oncol Res. (2017) 25:1269. doi: 10.3727/096504017X14850151453092 28117027 PMC7841022

[39] Yu B Ding Y Liao X Wang C Wang B Chen X . Overexpression of TONSL might be an independent unfavorable prognostic indicator in hepatocellular carcinoma. Pathol Pract. (2019) 215:939–45. doi: 10.1016/j.prp.2019.01.044 30723051

[40] Zhuang Q Zhou T He C Zhang S Qiu Y Luo B . Protein phosphatase 2A-B55δ enhances chemotherapy sensitivity of human hepatocellular carcinoma under the regulation of microRNA-133b. J Exp Clin Cancer Res. (2016) 35:1–15. doi: 10.1186/s13046-016-0341-z 27074866 PMC4831140

[41] Thapa N Chwae Joon Y Yoo Ho K Won T-B Kang D Choi D . Exosomal delivery of TRAIL and miR−335 for the treatment of hepatocellular carcinoma (Review). Int J Mol Med. (2023) 51:3. doi: 10.3892/ijmm.2022.5206 36416311 PMC9728509

