# Corrigendum: Diagnostic performance of microRNAs for predicting response to transarterial chemoembolization in hepatocellular carcinoma: a meta-analysis

**DOI:** 10.3389/fonc.2025.1578891

**Published:** 2025-03-06

**Authors:** Tianyi Huang, Jing Chen, Lu Zhang, Rui Wang, Yiheng Liu, Cuihua Lu

**Affiliations:** ^1^ Department of Gastroenterology, Affiliated Hospital of Nantong University, Medical School of Nantong University, Nantong, China; ^2^ Medical School of Nantong University, Nantong, China

**Keywords:** miRNAs, TACE, diagnostic accuracy, meta-analysis, HCC

In the published article, there was an error in affiliations 1, 2. Instead of “TianYi Huang^1,†^, Jing Chen^1,†^, Lu Zhang ^1,†^, Rui Wang ^1,†^, YiHeng Liu^1,†^, CuiHua Lu^1,2,†*^”, it should be “TianYi Huang^1,2 †^, Jing Chen^1,2 †^, Lu Zhang ^1,2 †^, Rui Wang^1,2 †^, YiHeng Liu^1,2 †^, CuiHua Lu^1,2 †*^”.

Furthermore, there was an error in the affiliations 1, 2. Instead of 1 Medical School of Nantong University, Nantong, China and 2 Medical School of Nantong University, Affiliated Hospital of Nantong University, Nantong, China, it should be 1 Department Gastroenterology, Affiliated Hospital of Nantong University, Medical School of Nantong University, Nantong, China and 2 Medical School of Nantong University, Nantong, China.

The authors apologize for this error and state that this does not change the scientific conclusions of the article in any way. The original article has been updated.

